# CRISPR-Cas and catalytic hairpin assembly technology for target-initiated amplification detection of pancreatic cancer specific tsRNAs

**DOI:** 10.3389/fbioe.2023.1169424

**Published:** 2023-05-03

**Authors:** Jie Wu, Hongpan Xu, Fenghua Hu, Yiyue Jiang, Boyue Fan, Adeel Khan, Yifan Sun, Kaili Di, Xinrui Gu, Han Shen, Zhiyang Li

**Affiliations:** ^1^ Department of Clinical Laboratory, Nanjing Drum Tower Hospital Clinical College of Jiangsu University, Nanjing, China; ^2^ State Key Laboratory of Bioelectronics, National Demonstration Center for Experimental Biomedical Engineering Education (Southeast University), School of Biological Science and Medical Engineering, Southeast University, Nanjing, China; ^3^ Department of Clinical Laboratory, Nanjing Drum Tower Hospital, Affiliated Hospital of Medical School, Nanjing University, Nanjing, China

**Keywords:** pancreatic cancer, tsRNAs, CRISPR-Cas12a, catalytic hairpin assembly, target-initiated amplification

## Abstract

Transfer RNA-derived small RNAs (tsRNAs) tRF-LeuCAG-002 (ts3011a RNA) is a novel class of non-coding RNAs biomarker for pancreatic cancer (PC). Reverse transcription polymerase chain reaction (RT-qPCR) has been unfit for community hospitals that are short of specialized equipment or laboratory setups. It has not been reported whether isothermal technology can be used for detection, because the tsRNAs have rich modifications and secondary structures compared with other non-coding RNAs. Herein, we have employed a catalytic hairpin assembly (CHA) circuit and clustered regularly interspaced short palindromic repeats (CRISPR) to develop an isothermal and target-initiated amplification method for detecting ts3011a RNA. In the proposed assay, the presence of target tsRNA triggers the CHA circuit that transforms new DNA duplexes to activate collateral cleavage activity of CRISPR-associated proteins (CRISPR-Cas) 12a, achieving cascade signal amplification. This method showed a low detection limit of 88 aM at 37 °C within 2 h. Moreover, it was demonstrated for the first time that, this method is less likely to produce aerosol contamination than RT-qPCR by simulating aerosol leakage experiments. This method has good consistency with RT-qPCR in the detection of serum samples and showed great potential for PC-specific tsRNAs point-of-care testing (POCT).

## Highlights

Catalytic hairpin assembly (CHA) circuit and clustered regularly interspaced short palindromic repeats (CRISPR) (CHA-CRISPR) is a simple, isothermal, ultrasensitive, and target-initiated amplification method to detect ts3011a RNA, a novel class of non-coding RNAs biomarkers for pancreatic cancer (PC).
Based on CHA-CRISPR method, this assay achieved simple, isothermal, ultrasensitive and target-initiated amplification of ts3011a RNA with a low detection limit of 88 aM in 2 h at 37 °C.
Moreover, it was demonstrated for the first time that, this method is less likely to produce aerosol contamination than Reverse transcription polymerase chain reaction (RT-qPCR) by simulating aerosol leakage experiments. This method has good consistency with RT-qPCR in detection of serum samples and showed great potential for PC specific tsRNAs point-of-care testing (POCT). Its diagnostic performance was found to be similar to that of RT-qPCR, and it is more suitable in community hospitals without precision instruments and PCR testing standard environments.

## 1 Introduction

Pancreatic cancer (PC) is the cancer with the lowest 5-year survival rate (Siegel et al., 2022). Because of the lack of markers for early diagnosis, most patients are in an advanced stage when they are diagnosed (Pereira et al., 2020). As a kind of transfer RNA-derived small RNAs (tsRNAs) (Li et al., 2018a; Jin and Guo, 2019), tRF-Leu-CAG-002 (ts3011a) was found to be a potential diagnostic marker for early pancreatic cancer (Jin et al., 2021). TsRNAs are newly discovered non-coding RNA that account for the vast majority of total small RNA in mammalian tissues and cells, and their existence ratios are much higher than that of the miRNAs (Zhang et al., 2014). The concentrations of ts3011a were between pM and fM in the clinically relevant ranges (Jin et al., 2021). Therefore, the development of point-of-care testing (POCT) technology using tsRNA as a marker will make it possible to carry out early pancreatic cancer screening in the community.

At present, reverse transcription PCR (RT-qPCR) is a commonly used non-coding RNA detection technology (Liu et al., 2012; Li et al., 2018b; Xiong et al., 2018). This technique requires sophisticated equipment and specialized laboratories that are susceptible to aerosol contamination by leakage of high-concentration amplification products (Chen et al., 2005; Ye et al., 2019). Because tsRNAs have more abundant modifications and secondary structures than other non-coding RNA (Li et al., 2018a), it has not been reported whether the isothermal technology can be used for detection. The combination of clustered regularly interspaced short palindromic repeats associated (CRISPR-Cas) system with target isothermal amplification methods, such as reverse transcription loop-mediated isothermal amplification (RT-LAMP) (Rezaei et al., 2021) and reverse transcription recombinase polymerase amplification (RT-RPA) (Chen et al., 2022a), can detect non-coding RNA such as miRNA and lncRNA without precision instruments, but they are prone to aerosol pollution (Borst et al., 2004). Target-initiated amplification methods like hybridization chain reaction (HCR) (Wang et al., 2019) and catalytic hairpin assembly (CHA) (Chen et al., 2022b) combined with CRISPR-Cas are not easy to cause aerosol pollution in theory, and CHA (Peng et al., 2020; Zeng et al., 2022) technology takes less time than HCR technology (Xing et al., 2020; Zhou et al., 2022a), making it more suitable for POCT diagnosis. However, whether CHA can be used for tsRNAs detection, and whether it can produce aerosol pollution needs further experimental verification.

**SCHEME 1:**
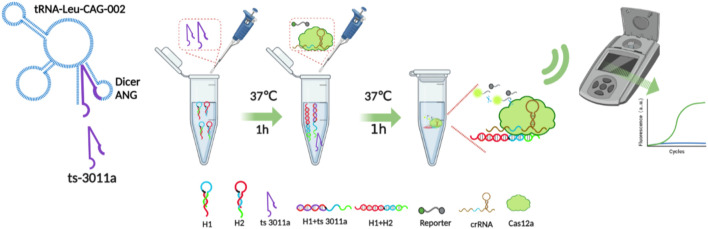
Schematic of fluorescence assay for tsRNA based on CHA reaction and CRISPR-Cas12a system.

Herein, we illustrated a simple method by coupling CRISPR/Cas12a and CHA to realize the detection of ts3011a RNA. In our experiment, two hairpins from the CHA circuit were triggered by tsRNAs and transformed into new DNA duplexes. The new DNA duplexes consisted of protospacer adjacent motif (PAM) and protospacer sequence that can be recognized by Cas12a/CRISPR RNA (crRNA) complexes. Therefore, the collateral cleavage activity of CRISPR/Cas12a was initiated, and cascade signal amplification was realized through the cleavage events of fluorescent ssDNA reporter molecules (Scheme 1). Based on the above principle, the experimental conditions were optimized, and a simulated aerosol leakage experiment was designed to verify whether the method would produce aerosol pollution. And for the detection of pancreatic cancer, the diagnostic efficiency was evaluated.

## 2 Experiment section

### 2.1 Materials and instruments

NaCl, KCl, MgCl_2_, and glycerol were purchased from Macklin Biochemical Co., Ltd., Shanghai, China. Tris hydrochloric acid (TrisHCl) was purchased from Dalian Meilun Biological Technology Co., Ltd., Suzhou, China. Dithiothreitol (DTT) was purchased from SaiGuo Biotech Co., Ltd., Guangzhou, China. Lachnospiraceae bacterium (LbCas12a) was purchased from GenScript Biotech Co., Ltd., Nanjing, China, and the protein was stored at −80 °C until later use. Agarose powder, ×10 Trizma base boric acid ethylene diamine tetra acetic acid (TBE) buffer, and ×6 Loading buffer were obtained from Sangon Biotech, Shanghai, China. All oligonucleotides (Supplementary Table S1) and diethylpyrocarbonate (DEPC) treated water were obtained from Sangon (Shanghai, China). All chemical reagents used in the study were of analytical grade, and all the solutions were prepared with DEPC-treated water. Human pancreatic carcinoma cells (PANC-1), and human pancreatic duct cells (hTERT-HPNE) were bought from Zhong Qiao Xin Zhou Biotechnology Co., Ltd. (Shanghai, China). Cell culture medium, fetal bovine serum (FBS), trypsin,penicillin–streptomycin, and dulbecco’s modified eagle medium (DMEM) were bought from Invitrogen (Gibco, United States). TRIzol RNA isolation reagents were bought from Thermo Fisher Scientific (Wilmington, United States). Primer Script ™ RT reagent Kit and TB Green Premix Ex Taq ™ II qPCR Kit were provided by Takara Biomedical Technology Co., Ltd. (Beijing, China). The primers (Supplementary Table S1) were designed and synthesized by Sangon (Shanghai, China). The humidified incubator was used for cell culture (Thermo Fisher Scientific, United States). The annealing buffer used in the annealing step was bought from Beyotime Biological Technology Co., Ltd. (Shanghai, China) and Gel Imaging System used Tanon 2500R for photographing (Shanghai, China). DNA annealing process, RT-qPCR, and fluorescence intensity (FL) testing were carried out on C1000™ Thermal Cycler (Bio-Rad Laboratories, Inc., United States). A cyclone air sampler (ASP-200p, Shenzhen Lemniscare Medical Technology Co., Ltd., China) was used for sampling.

### 2.2 Performing tsRNA-triggered CHA circuits in solution

The structures of H1 and H2 were predicted by https://sg.idtdna.com/calc/analyzer. The H1 and H2 were annealed at 95 °C for 5 min and then gradually cooled down at 4 °C for 1 h to obtain the desired stem-loop structure using a thermal cycler respectively. The CHA reaction buffer consisted of 5 mM KCl, 20 mM Tris-HCl, and 140 mM NaCl, pH 7.5. A 10 μL CHA reaction system contained 2 μM H1, 2 μM H2, and different concentrations of tsRNA to conduct the reaction at 37 °C for 1 h. Besides, we used fluorescence quenching H1 to detect time-dependent fluorescent signals of CHA in the presence of different targets, mainly including ts3011a, tRF-3, tRF-4, and tRF-10 (Supplementary Figure S1).

### 2.3 Gel electrophoresis analysis

Native PAGE analysis was performed to characterize the CHA products. A measure of 2 µL of ×6 loading buffer was mixed with 10 µL of each sample, and then 10 µL of the above-mixed solution was loaded into a 12% PAGE, running at 100 V for 2 h in ×1 TBE buffer. After that, the gel was stained for 10 min in 50 mL ×1 TBE buffer with 5 μL ×10,000 Gel-Red nucleic acid gel stain. The gels were then photographed.

### 2.4 Detection of ts3011a with CRISPR-CHA assay

Subsequently, 1 μL of the above CHA reaction solution was added into the cleavage buffer (5% glycerol, 1 mM DTT, 5 mM MgCl_2_, 20 mM tris HCl, and 100 mM KCl, pH 7.5) containing 400 nM CRISPR-Cas12a, 200 nM crRNA, and 1 μM FAM-TTATT-BHQ1 reporter molecules (FQ), making a final volume of 10 μL. The fluorescence emission measurements were recorded at 518 nm in the thermal cycler at 37 °C for 1 h. The stem length, CHA reaction time, temperature, and concentration of crRNA and Cas enzyme in 10 μL volume were optimized.

### 2.5 Cell culture and ts3011a extraction

PANC-1 and hTERT-HPNE were grown in DMEM supplemented with 10% FBS, 1%penicillin–streptomycin (100 U/mL penicillin and 100 μg/mL streptomycin) and cultured at 37 °C in a humidified incubator containing 95% air and 5% CO_2_. Total RNA was extracted using TRIzol RNA isolation reagents. cDNA was prepared by Primer Script™ RT reagent Kit. Each measure of 10 μL of reaction system included 2 μL of primer script buffer (×5) and 0.5 μL of primer script enzyme, which were mixed with 0.5 μL primer, 2 μL of total RNA, and 5 μL of DEPC treated water. The reactions were then incubated at 42 °C for 15 min, 85 °C for 5 s, and cDNA was stored at −20 °C until further use.

### 2.6 RT-qPCR analysis of ts3011a expression

RT-qPCR analysis of ts3011a expression was conducted by qPCR Kit. The whole 25 μL of reaction volume contained 12.5 μL of ×2 Premix Ex Taq II, 1 μL of both forward and reverse primers, 2 μL of cDNA, and 8.5 μL of DEPC treated water. The reaction was conducted using a thermal cycler according to the guidelines of the manufacturer (at 95 °C for 30 s, followed by 40 cycles of 95 °C for 5 s and 60 °C for 30 s). The relative expression of cell samples was evaluated by referring to the expression of the mir39 (external control for miRNAs) using the ^2–ΔΔ^ Ct method (Supplementary Table S2).

### 2.7 Simulated aerosol detection experiment

CHA, CHA-CRISPR, and RT-qPCR were performed with 1 nM ts3011a, respectively, to simulate the possible release of aerosol after CHA, CHA-CRISPR, and RT-qPCR reaction. The closed hood was opened to ventilate for 0.5 h. The air sampler with 2 mL of sampling liquid (CHA or RT-qPCR reaction buffer) was put into the closed hood. The 8-strip tubes with CHA or CHA-CRISPR or RT-qPCR products were then put into the closed hood and uncapped. The closed-hood lead was closed and left for 5 min. The sampler was then turned on for 15 min with an air velocity of 200 L/min (Chen et al., 2023). CHA or CHA-CRISPR or RT-qPCR respectively were then performed using 1 μL of the sample liquid according to steps 2.4 and 2.6. After each aerosol collection experiment, the inner wall of the closed cover and the sampler were wiped twice with water and 75% alcohol.

### 2.8 Quantification of ts3011a in serum samples with CHA-CRISPR

All the pancreatic cancer serum samples and healthy serum samples were collected from consented patients at the Nanjing Drum Tower Hospital (Nanjing, China) from January to October 2022. Approval was obtained from our hospital ethics committee. 200 μL of serum samples were centrifuged at 1700 g for 20 min at 4 °C. The TRIzol RNA isolation reagents were used to extract the total RNA. 1 μL of total RNA was added to the CHA-CRISPR system to validate its potential feasibility.

## 3 Results and discussion

### 3.1 Principle of CHA-CRISPR

**FIGURE 1:**
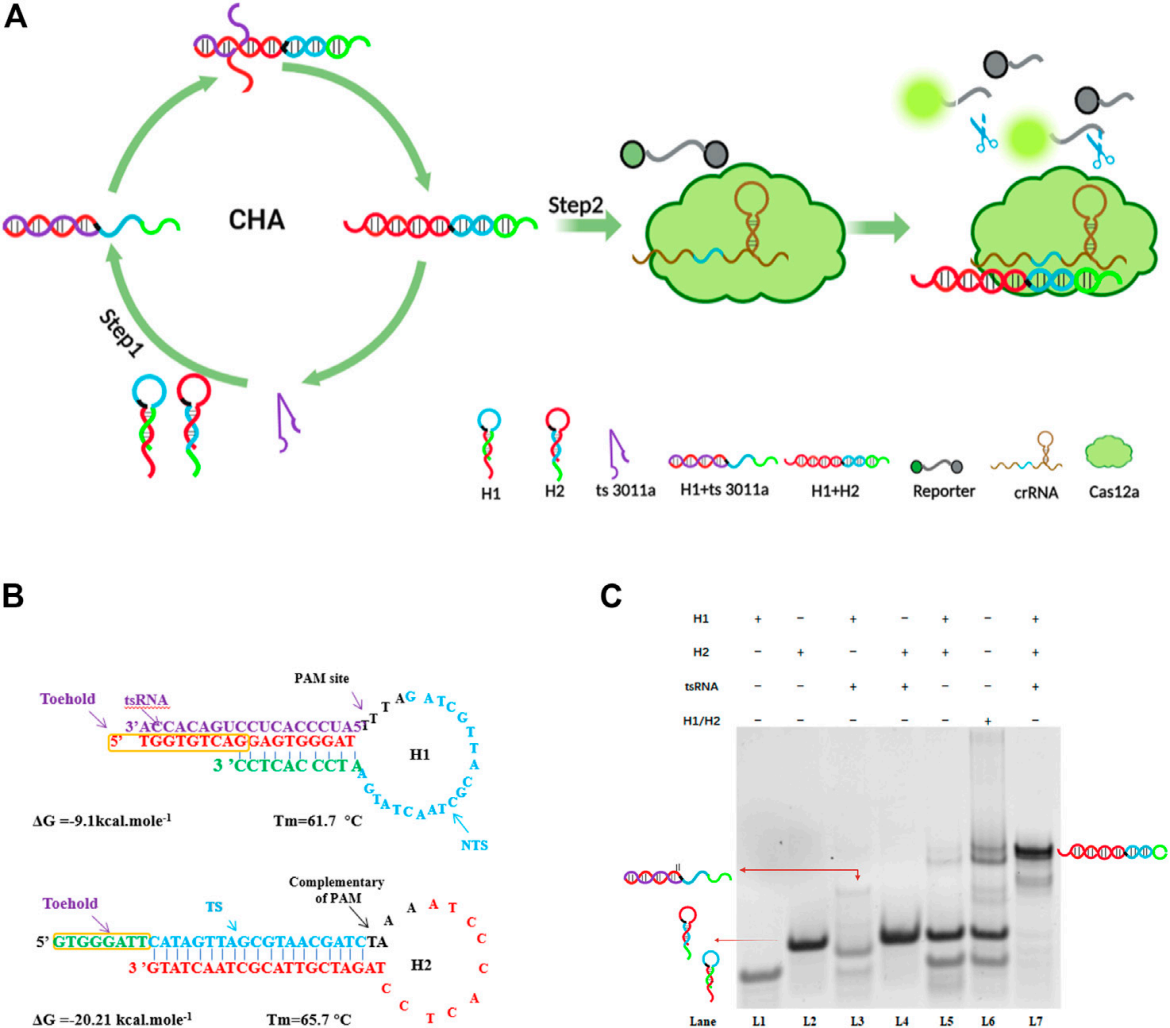
**(A)** Specific demonstration of CHA-CRISPR mechanism for detecting tsRNAs. **(B)** Detailed sequences of H1 and H2 in the CHA circuit for detection of tsRNAs. TS, target sequence; NTS, non-target sequence. **(C)** Electrophoresis analysis of the CHA system; H1 (2 μM); H2 (2 μM); ts3011a RNA (100 nM). H1/H2 were not annealed, H1 + H2 were annealed.

In the first step of amplification, CHA cycles contained the following three main elements: two hairpins (H1 and H2) and target ts3011a RNAs as initiators (Figure 1A). Both designed H1 and H2 had a stem-loop DNA containing majority complementary regions and an 8-nt 5′overhang as a toehold, which remained at metastable state that coexisted in the absence of ts3011a RNAs. As illustrated in Figure 1B, H1 contained the sequence “TGG​TGT​CAG​GAG​TGG​GAT”, which was perfectly complementary to the sequence of the target tsRNA, and the loop region contained the 5′-TTTA-3′ protospacer adjacent motif (PAM) of Cas12a, as well as a non-target strand (NTS) “TTT​AGA​TCG​TTA​CGC​TAA​CTA​TGA”. The length of NTS was 20 nt (Feng et al., 2021; Kaminski et al., 2021). The target tsRNA binding to the exposed toehold domain of H1 triggered a branch migration, generating an H1-tsRNA intermediate through domain hybridization. The exposed toehold domain “ATCCCACTCC” in the H1/tsRNA complex further triggered strand displacement on domain “GTG​GGA​TTC​ATA​GTT​AGC​GTA​ACG​ATC​TAA​AAT​CCC​ACT​CC” of H2, which led to the dynamic assembly of H1 and H2, forming mass H1/H2 duplexes and releasing target ts3011a RNAs (Figure 1A). The Cas12a proteins, crRNA, and FQ were introduced in the second step of amplification to mix with H1/H2 duplexes. The Cas12a/crRNA recognized the PAM-TTTA and bound to the protospacer sequence of H1/H2 duplexes, resulting in the activation of collateral cleavage activity of Cas12a to cleave the reporter molecules, hence, generating intense fluorescence signals for tsRNA quantification (Figure 1A).

To verify the feasibility of the assay, we conducted gel electrophoresis (Figure 1C). Only when H1, H2, and target ts3011a RNAs coexisted in the reaction solution, did obvious hybridization bands with higher molecular weight appear as lane 7 (Figure 1C), suggesting the formation of H1/H2 duplexes. Moreover, hybridization bands were the same size as H1/H2 duplexes when H1 and H2 did not form hairpins, as seen in lane 6 (Figure 1C). The H1 and H2 did form hairpins after annealing, remaining at a metastable state which coexisted as shown in lane 5 (Figure 1C). A negligible band of the H1/H2 duplex was observed in the absence of ts3011a ( Figure 1C, lane 5), which indicated low background leakage in the CHA circuit, as demonstrated in previous reports (Wang et al., 2020; Zhou et al., 2022b). An obvious band for ts3011a RNAs/H1 duplex was observed in lane 3, indicating effective initiation of the CHA cycles by ts3011a RNAs. To show the effect of target concentration on H1/H2 and H1/ts3011a complex formation, we added FQ-H1 experiments (Supplementary Figure S1 **)**. When the target concentration was 10 nM, the fluorescence increased significantly, and with the decrease of the target concentration, the fluorescence intensity decreased (Supplementary Figure S1A **)**. Besides, CHA has good specificity and can distinguish homologous RNA (Supplementary Figure S1B **)**.

### 3.2 Experimental optimization

**FIGURE 2:**
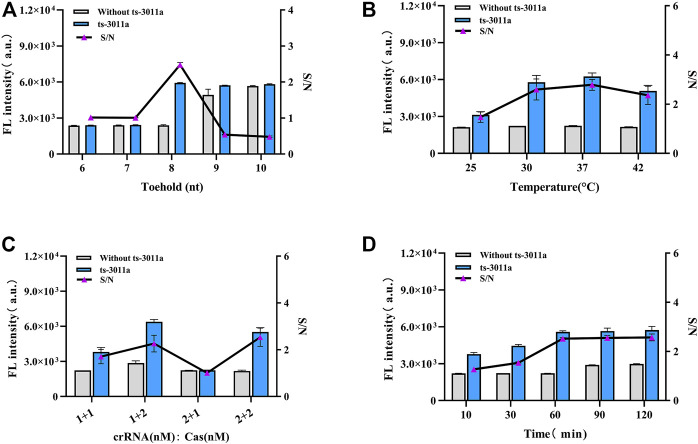
Optimization of experimental parameters. **(A)** Different base numbers of toeholds for H1 and H2. **(B)** Cleavage temperature. **(C)** The concentrations of crRNA and Cas12a, 1 + 1 (crRNA 200 nM, Cas12a 200 nM); 1 + 2 (crRNA 200 nM, Cas12a 400 nM); 2 + 1 (crRNA 400 nM, Cas12a 200 nM); 2 + 2 (crRNA 400 nM, Cas12a 400 nM). **(D)** CHA reaction time. Concentrations of H1 and H2 from A to D groups were 2 μM; ts3011a RNA (10 pM). Data are presented as mean ± S.D from three replicates measurements.

The stem length was vital in the design of H1 and H2 (Shen et al., 2020; Cui et al., 2021). The complementary stem length was too long to facilitate the opening of the hairpin, and when too short, complementary bases have poor specificity. As investigated from 6 to 10 nt toehold length of H1 and H2 in the presence of 1 nM ts3011a RNAs, the FL intensity results showed that 8-nt 5′overhang toeholds were best for initiation of reaction as shown in Figure 2A. The toehold length (8 nt) had the lowest background in electrophoresis (Supplementary Figure S2). And 37 °C was the best temperature (Figure 2B). In addition, the concentration optimization results for crRNA and Cas enzyme showed that the fluorescence intensity increased with the efficiency of the Cas enzymes. There was maximum S/N (signal/noise) at crRNA 200 nM and Cas 400 nM (Figure 2C). The results from CHA reaction time showed that the FL intensity increased significantly with increased hybridization time and the FL intensity gradually stabilized after 60 min (Figure 2D). Therefore, 60 min was selected as the best CHA reaction time.

### 3.3 Linear range and specificity of the proposed circuit

**FIGURE 3:**
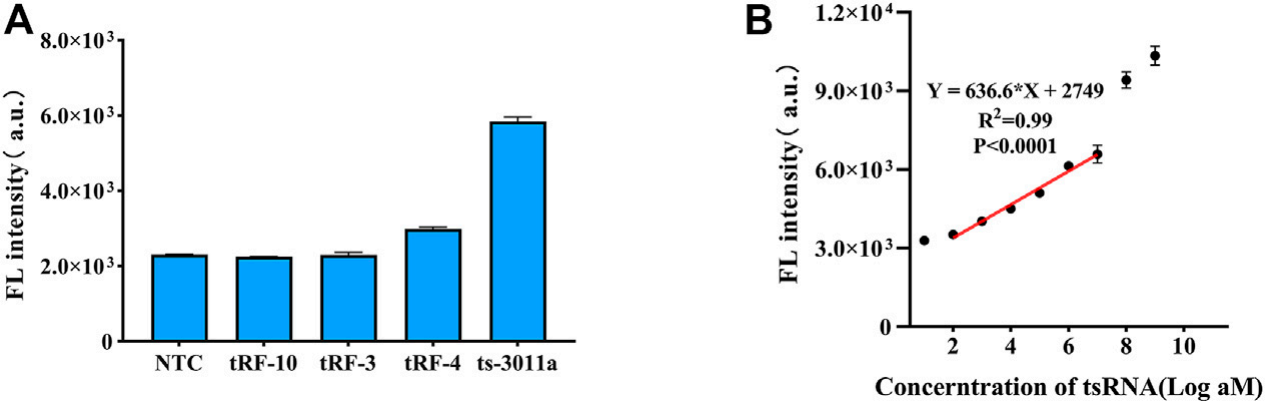
**(A)** Specificity of CHA-CRISPR detection of ts3011a (10 pM) against different kinds of tsRNAs (10 pM). **(B)** Linear plot for CHA-CRISPR vs. logarithm of ts3011a at 100 aM to 1 nM concentrations. NTC, No Template Control. Data are presented as mean ± S. D from three replicate measurements.

The detection of ts3011a RNAs by the DNA circuit should be sequence-specific, so we evaluated the other three kinds of tsRNA (sequences shown in Supplementary Table S1). Among them, the sequence of tRF-4 was similar to that of ts3011a RNAs, with 12 same nucleotides, thus showing a higher fluorescence detection signal (Figure 3A). However, it also confirmed that the CHA-CRISPR assay achieved highly specific detection of ts3011a RNAs.

The standard ts3011a RNA samples were diluted with DEPC and quantified by RT-qPCR (Supplementary Figure S3). Fluorescence signals were collected from 0 to 1 nM in 1 h cleavage of excessive FQ reporter molecules. With the increase of target concentration, the fluorescence intensity also increased (Supplementary Figure S4). As illustrated in Figure 3B, an obvious linear relationship of CHA-CRISPR ranging from 100 aM to 10 pM (*R* ^2^ = 0.99), with a limit of detection (LOD) of 88 aM (3δ/S, *δ* indicating the standard deviation of the blank control and S indicating the slope of calibration curve) (Choengchan et al., 2006). The sensitivity of the proposed method was comparable to the RT-qPCR (Supplementary Figure S3).

### 3.4 Simulated aerosol detection experiment

**FIGURE 4:**
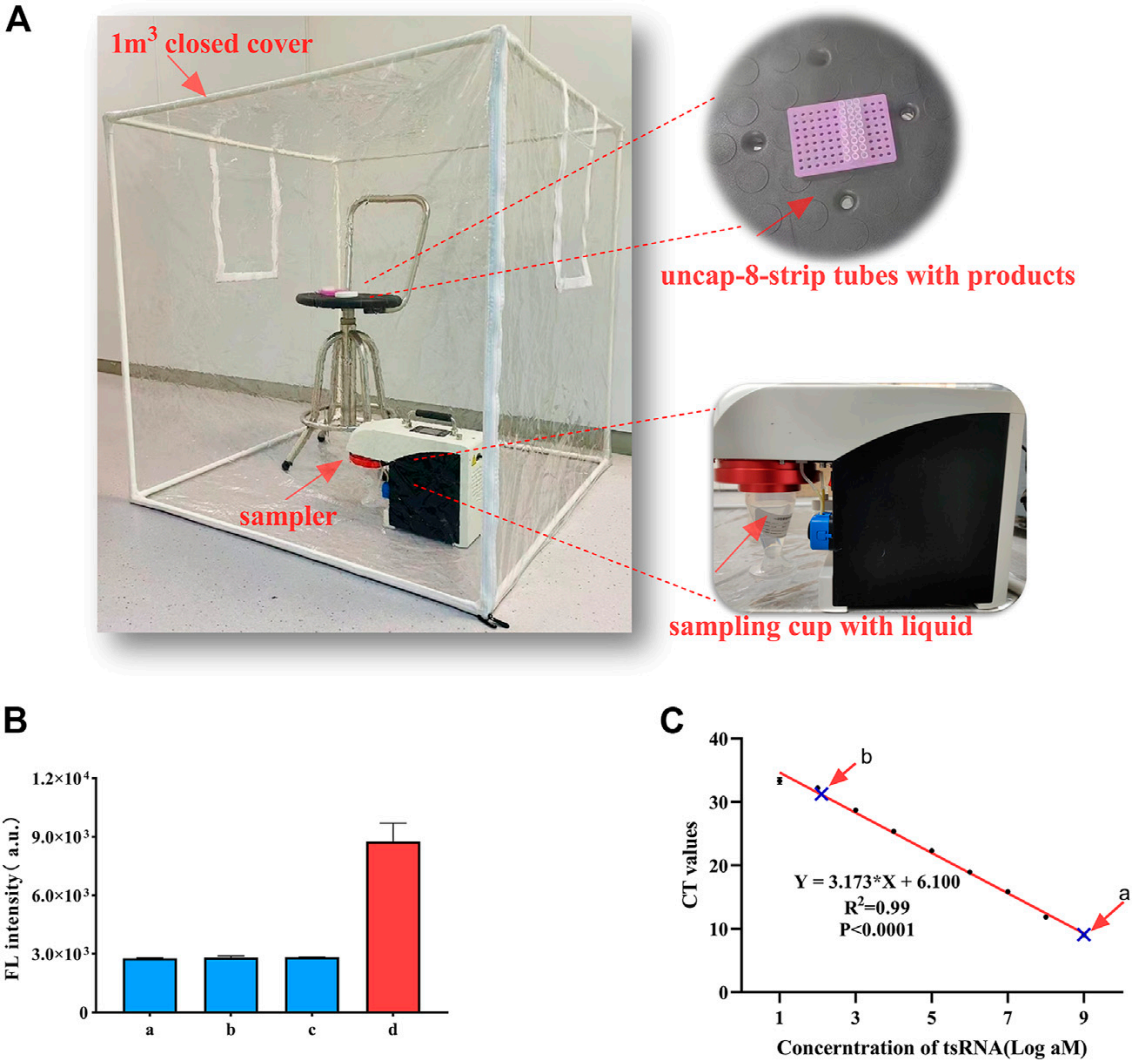
**(A)** Sampling scene (including the sampler device and samples). **(B)** Aerosol detection of CHA-CRISPR. a, blank control (without ts3011a); b, collected sampling liquid of CHA products; c, collected sampling liquid of CHA-CRISPR products; d, ts3011a (1 nM). **(C)** Aerosol detection of RT-qPCR. a, ts-3011a (1 nM); b, collected sampling liquid of RT-qPCR products. Data are presented as mean ± S.D. from three replicate measurements.

The detection device is shown in Figure 4A. The aerosol was generated by a momentary shock, and we chose to open the lids for 5 min because the aerosol was still floating in the air and was not completely settled to be captured by the sampler. The results showed that the fluorescence generated by the collected sampling liquid of 1 nM ts3011a CHA-CRISPR and CHA products did not cause a significant increase compared to blank control (Figure 4B). RT-qPCR was performed with 1 nM ts3011a collected sampling liquid of RT-qPCR products can cause amplified contamination of 0.21 fM (Figure 4C; Supplementary Table S3). These results suggested that the CHA-CRISPR can avoid aerosol contamination caused by amplified products.

### 3.5 Quantification of ts-3011a in cells and serum samples with CHA-CRISPR

**FIGURE 5:**
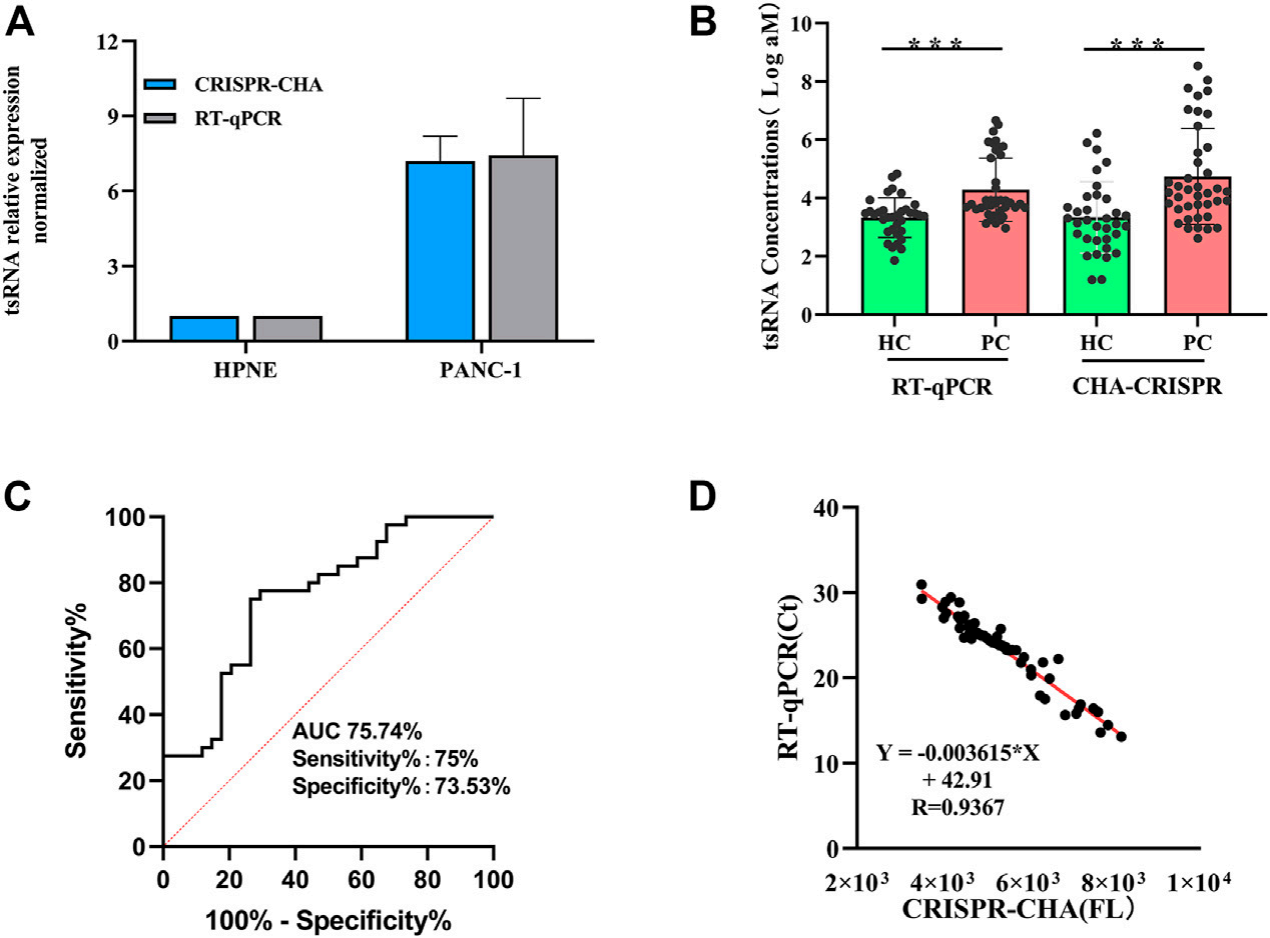
Diagnostic efficiency evaluation results. **(A)** Expression levels of ts3011a in cells using CHA-CRISPR and RT-qPCR. **(B)** Serum ts3011a expression level was detected by CHA-CRISPR and RT-qPCR. HC, healthy control (including 40 PC patients and 34 control individuals). *p* < 0.001. **(C)** Receiver Operating Characteristic (ROC) curves for CHA-CRISPR for ts3011a concentrations in serum samples (including 40 PC patients and 34 control individuals). **(D)** Correlation of CHA-CRISPR assay fluorescence intensity with clinical RT-qPCR Ct values. Data are presented as mean ± S. D from three replicate measurements.

The expression levels of ts3011a RNA in PANC-1 and hTERT-HPNE were analyzed with CHA-CRISPR and RT-qPCR (Supplementary Table S2). The quantification results showed that the CHA-CRISPR was consistent with RT-qPCR (Figure 5A). The level of ts3011a RNAs in the serum samples was also detected (Figure 5B). These results showed that the levels of ts3011a RNAs from PC patients were much higher than those from healthy individuals, which was consistent with recently reported results (Jin et al., 2021). Besides, the CHA-CRISPR demonstrated a significantly superior diagnostic accuracy for early PC patients, with an area under the curve (AUC) of 0.7574, exhibiting 75% sensitivity and 75.53% specificity (Figure 5C), which is consistent with RT-qPCR (which showed the sensitivity of 66.67% and specificity of 77.78%, Supplementary Figure S5). From Figure 5D **,** the fluorescence intensity of the CHA-CRISPR assay demonstrated a good agreement with the Ct values of RT-qPCR in the detection of serum samples (R = 0.9367).

## 4 Conclusion

In summary, based on CHA and CRISPR-Cas12a, we have first developed a target-initiated amplification detection of pancreatic-cancer-specific tsRNAs. Under simulated experimental conditions, aerosol pollution was not produced by products of CHA-CRISPR with 1 nM tsRNA. The detection was achieved at 37 °C within 2 h, with a detection limit of 88 aM. Its diagnostic performance was found to be similar to that of RT-qPCR, and it is more suitable in community hospitals without precision instruments and PCR testing standard environments.

## Data Availability

The original contributions presented in the study are included in the article/Supplementary Material, further inquiries can be directed to the corresponding authors.
