# Supplementary material for: CRISPR-Cas and catalytic hairpin assembly technology for target-initiated amplification detection of pancreatic cancer specific tsRNAs
Source: Front Bioeng Biotechnol. 2023 May 3;11:1169424. doi: 10.3389/fbioe.2023.1169424 (PMC10188930; doi:10.3389/fbioe.2023.1169424)
Supplement: Supplementary file 1 [file DataSheet1.docx]

Supplementary Material

**CRISPR-Cas and Catalytic Hairpin Assembly Technology for Target-Initiated Amplification Detection of Pancreatic Cancer Specific tsRNAs**

**Jie Wu ^1^ †, Hongpan Xu ^1^ †, Fenghua Hu ^1^ †, Yiyue Jiang ^1^, Boyue Fan ^1^,** **Adeel Khan ^2^, Yifan Sun ^1^, Kaili Di ^3^, Xinrui Gu ^3^,** **Han Shen^1 *^, Zhiyang Li ^1 *^**

^1^ Nanjing Drum Tower Hospital Clinical College of Jiangsu University, Nanjing, China

^2^ State Key Laboratory of Bioelectronics, School of Biological Science and Medical Engineering, National Demonstration Center for Experimental Biomedical Engineering Education (Southeast University), Southeast University, Nanjing, China

^3^ Department of Laboratory Medicine, Nanjing Drum Tower Hospital, The Affiliated Hospital of Nanjing University Medical School, Nanjing, China

**Correspondence:** [lizhiyangcn@qq.com](mailto:lizhiyangcn@qq.com); [shenhan10366@sina.com](mailto:shenhan10366@sina.com).

†These authors contributed equally to this work and share first authorship.

*****Corresponding Author: Zhiyang Li*; Han Shen *.

# Supplementary Figures and Tables

## Supplementary Figures


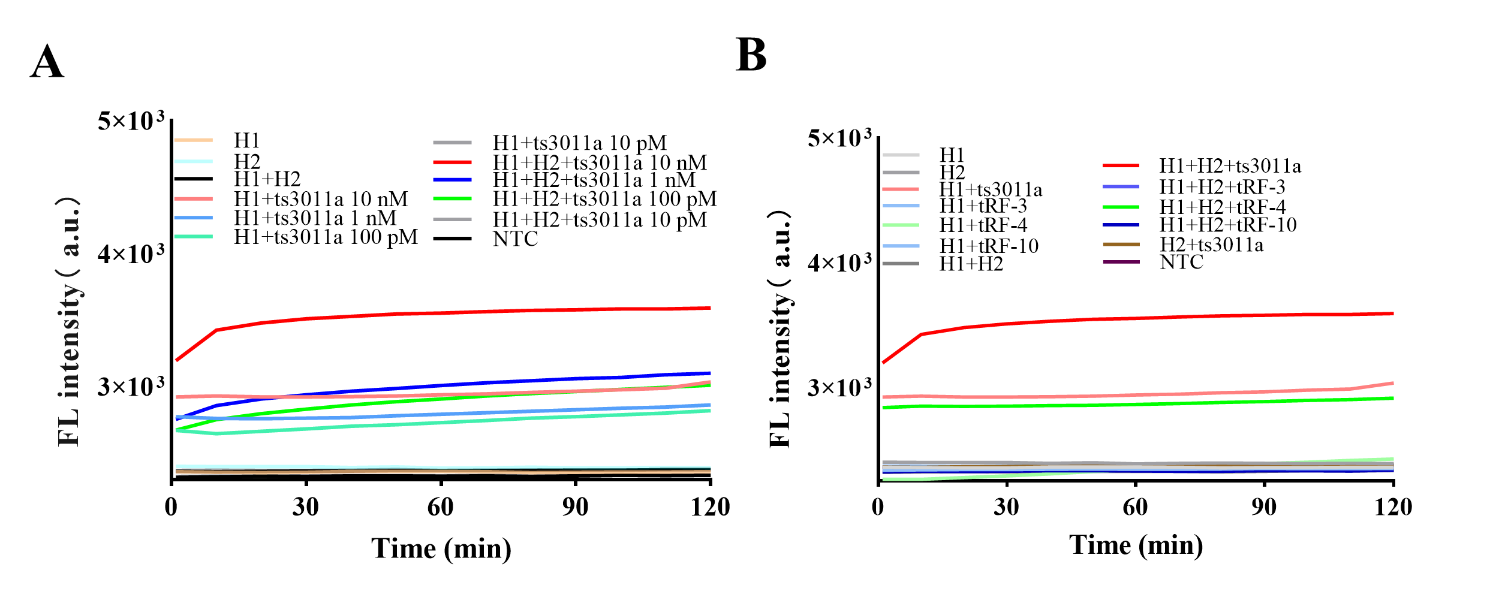


**Supplementary Figure 1** (A) Time-dependent fluorescent signals of CHA in the presence of ts3011a at 10 pM to 10 nM concentrations. (B) Time-dependent fluorescent signals of CHA in the presence of different targets, including H1; H2; H1+ts3011a; H2+ ts3011a; H1+H2; H1+tRF-3; H1+H2+tRF-3; H1+tRF-4; H1+H2+tRF-4; H1+tRF-10; H1+H2+tRF-10; H1+H2+ ts3011a and NTC. H1-FQ, 2 μM; H2, 2 μM; ts3011a, tRF-3, tRF-4, and tRF-10, 10 nM.


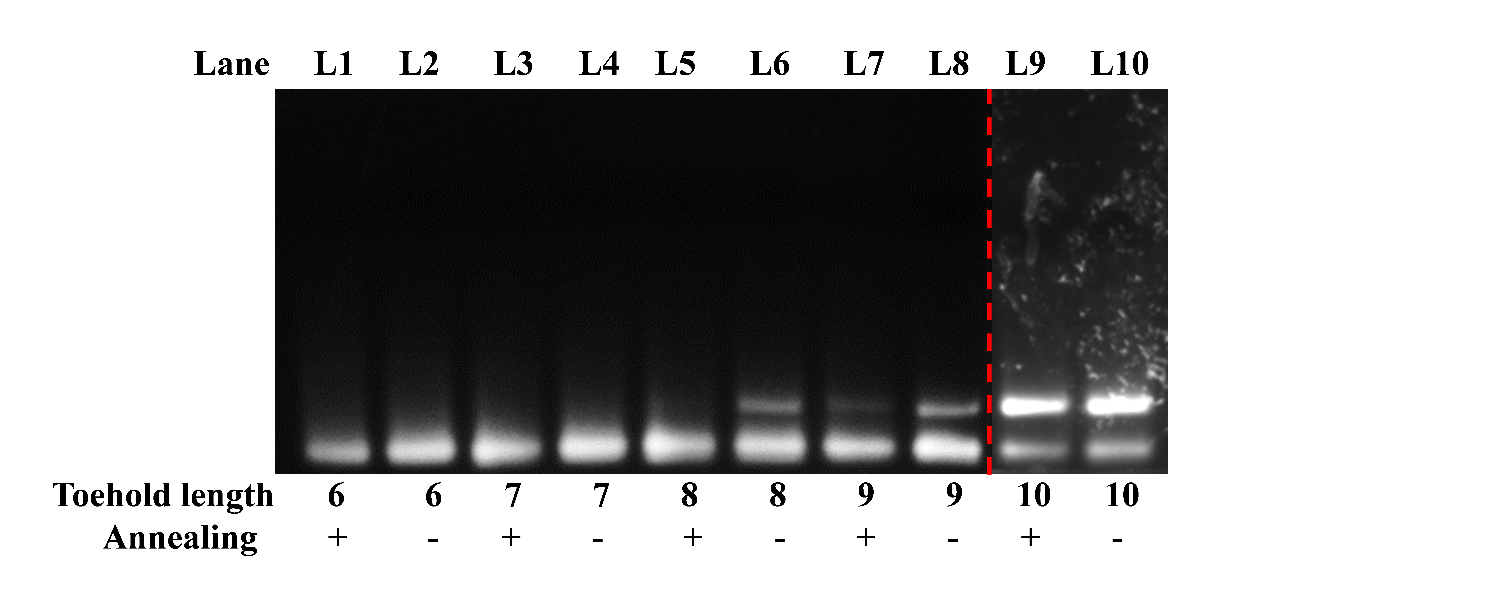


**Supplementary Figure 2** 3% agarose gel electrophoresis analysis of different toehold lengths of H1 and H2 in the CHA system. H1 and H2 were annealing (+) or non-annealing (-). H1(2 μM); H2 (2 μM); ts3011a RNA (100 nM). The red dotted line represented the splicing of two electrophoretic diagrams.

**
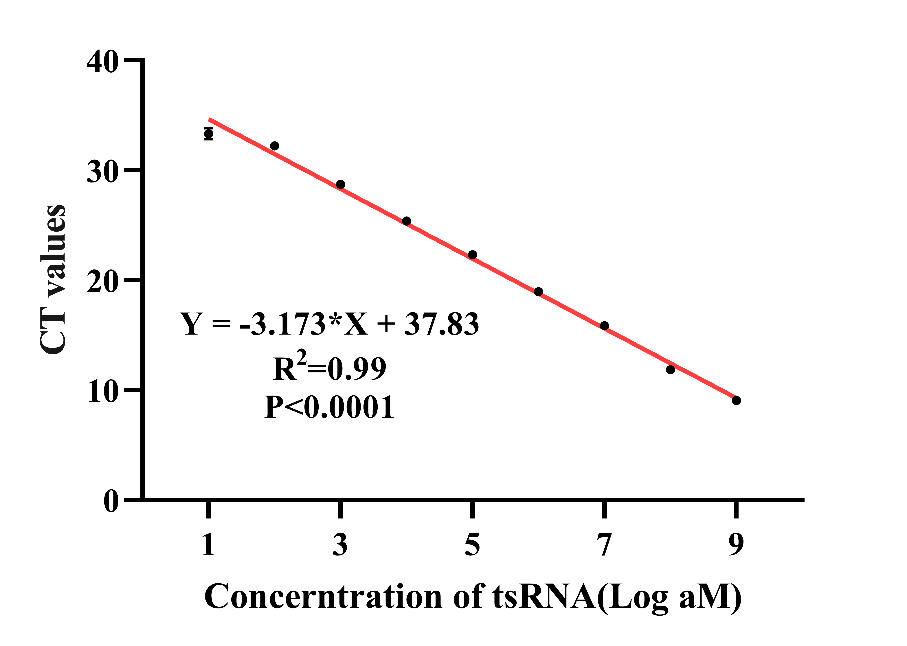
**

**Supplementary Figure 3** Linear plot of RT-qPCR logarithm of ts3011a at concentrations from 10 aM to 1 nM. Data are presented as mean ±S.D. from three replicate measurements.


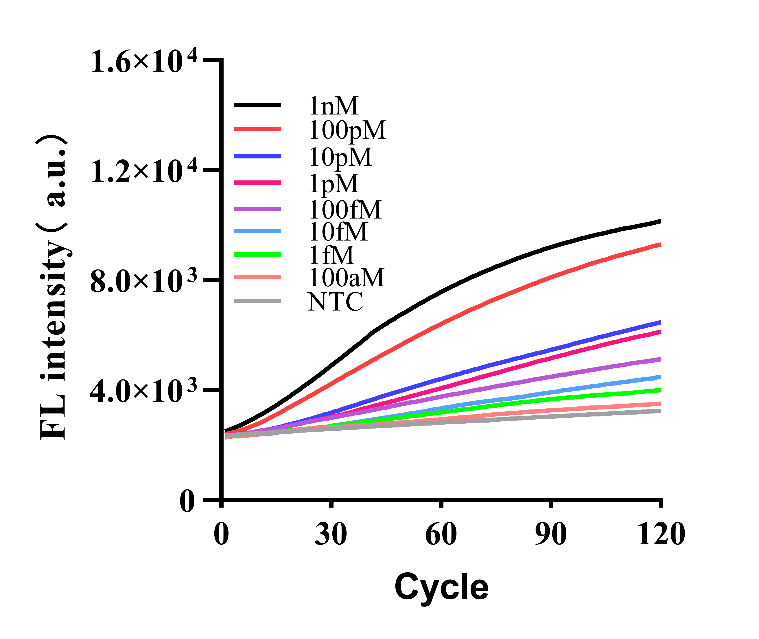


**Supplementary Figure 4** Amplification curves for CHA-CRISPR platform corresponding to various concentrations of ts3011a. 0 aM, 100 aM, 1 fM, 10 fM, 100 fM, 1 pM, 10 pM, 100 pM, and 1 nM. One cycle equals half a minute.


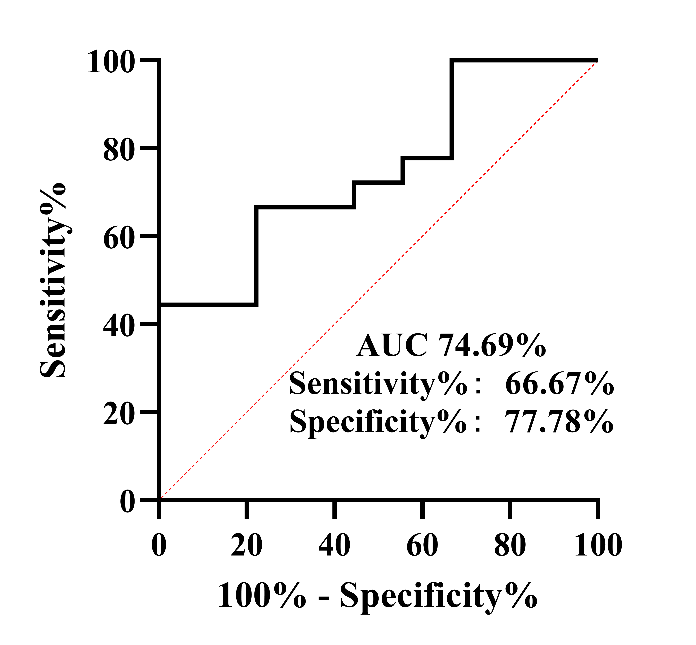


**Supplementary Figure 5** Receiver Operating Characteristic (ROC) curves for ts3011a in serum samples from PC patients versus healthy controls of RT-qPCR (including 40 PC patients and 34 control individuals).

## Supplementary Tables

| **Supplementary Table.1** Sequence of the oligonucleotides used for tsRNA assay in CHA-CRISPR | |
| --- | --- |
| **Oligonucleotides** | **Sequence (5’-3’)** |
| tsRNA(tRF-3a/tRF-LeuCAG-002) | AUCCCACUCCUGACACCA |
| tsDNA | ATCCCACTCCTGACACCA |
| ^a^crRNA | UAAUUUCUACUAAGUGUAGAUGAUCGUUACGCUAACUAUGA |
| ^bc^H1 | TGGTGTCAGGAGTGGGATTTTAGATCGTTACGCTAACTATGAATCCCACTCC |
| H2 | GTGGGATTCATAGTTAGCGTAACGATCTAAAATCCCACTCCTAGATCGTTACGCTAACTATG |
| 6SH1 | TGGTGTCAGGAGTGGGATTTTAGATCGTTACGCTAACTATGAATCCCACTCCTG |
| 6SH2 | GGGATTCATAGTTAGCGTAACGATCTAAAATCCCACTCCTGTAGATCGTTACGCTAACTATG |
| 7SH1 | TGGTGTCAGGAGTGGGATTTTAGATCGTTACGCTAACTATGAATCCCACTCCT |
| 7SH2 | TGGGATTCATAGTTAGCGTAACGATCTAAAATCCCACTCCTTAGATCGTTACGCTAACTATG |
| 9SH1 | TGGTGTCAGGAGTGGGATTTTAGATCGTTACGCTAACTATGAATCCCACTC |
| 9SH2 | AGTGGGATTCATAGTTAGCGTAACGATCTAAAATCCCACTCTAGATCGTTACGCTAACTATG |
| 10SH1 | TGGTGTCAGGAGTGGGATTTTAGATCGTTACGCTAACTATGAATCCCACT |
| 10SH2 | GAGTGGGATTCATAGTTAGCGTAACGATCTAAAATCCCACTTAGATCGTTACGCTAACTATG |
| tRF-3(tRF-1:28-His-GTG-1) | GCCGUGAUCGUAUAGUGGUUAGUACUCU |
| tRF-4(tRF-68:85-Ser-GCT-3-M4) | AUCCCAUCCUCGUCGCCA |
| tRF-10(tRF-57:76-Tyr-GTA-2-M2) | GAUUCCGGCUCGAAGGACCA |
| tsRNA(tRF-3a) forward primer | GCGCGATCCCACTCCTG |
| mir-39 forward primer | GCGCTCACCGGGTGTAAAT |
| FQ reporter  ^bc^H1-FQ | FAM-TTATT-BHQ  FAM- TGGTGTCAGGAGTGGGATTTTAGATCGTTACGCTAACTATGAATCCCACTCC -BHQ |
| aThe bases in green the sequence of the spacer. | |
| bThe bases in blue are the sequence of TS and NTS. | |
| cThe bases in orange are the sequence of PAM. | |

| **Supplementary Table.2** Average Ct values in RT-qPCR assay of ts3011a | | | |  |  |
| --- | --- | --- | --- | --- | --- |
| Note | Ct mir-39 | Ct ts3011a | ΔCt | -ΔΔCt | Fold change |
| PANC1 | 18.99 | 14.84 | -4.15 | -2.84 | 7.18 |
| hTERT-HPNE | 20.40 | 19.09 | -1.31 | 0.00 | 1.00 |

**Supplementary Table.3** Method comparison between RT-qPCR and CHA-CRISPR for detecting ts3011a

| Method | RT-qPCR | CHA-CRISPR |
| --- | --- | --- |
| Isothermal detection | No | Yes |
| Time (min) | 2 h | 2 h |
| LOD | 10 aM | 88 aM |
| Instrument requirements | High | Low |
| Easy to cause aerosol pollution | Yes | No |
